# P04.28. Implementing a mind-body medicine relaxation training program in an urban high school: changes in health behaviors, perceived stress, and anxiety

**DOI:** 10.1186/1472-6882-12-S1-P298

**Published:** 2012-06-12

**Authors:** H Wilson, M Scult, M Wilcher, R Chudnofsky, L Malloy, G Fricchione, H Benson, J Denninger

**Affiliations:** 1 Massachusetts General Hospital/Harvard Medical School, Boston, USA

## Purpose

The negative impact of excessive stress on adolescents is linked to increased substance abuse, violence, and depression. The Relaxation Response (RR), a physiologic response opposite to the fight-or-flight response, has been shown to be effective in treating anxiety/depression and reducing stress in high school students. Several studies have demonstrated that RR-based interventions that involve psychoeducation and teaching relaxation strategies are related to decreased anxiety and perceived stress in high school students. These interventions have previously been led by study staff. This study’s objective was to assess the feasibility of having high school teachers implement an RR curriculum with their own students.

## Methods

We taught teachers in a local public high school a RR curriculum that used diaphragmatic breathing, imagery, and relaxation training. Teachers then implemented this curriculum with students, and we assessed changes in self-reported teacher and student health behaviors, perceived stress, and anxiety using a pre-intervention/post-intervention survey. A four week follow-up survey was completed by students. All teachers and students received the intervention. Twelve teachers and 77 students completed the study.

## Results

Data analysis using paired sample t-tests found that after receiving the intervention teachers and students reported a significant increase in the use of positive health behaviors. Students also reported significantly less state and trait level anxiety after receiving the intervention. Significant results for students were maintained after a four week follow-up. All significant results were at p< .05 level when controlling for multiple comparisons.

## Conclusion

Further research is needed to replicate findings in a randomized-control study along with analysis of effects on academic performance and physical health. There are also policy implications. By documenting the efficacy of RR based interventions, an argument can be made to incorporate such training into teacher education and educational curricula to make a wide-spread impact on health and well-being.

